# Assessing the Quality of Mobile Health-Related Apps: Interrater Reliability Study of Two Guides

**DOI:** 10.2196/26471

**Published:** 2021-04-19

**Authors:** Jordi Miró, Pere Llorens-Vernet

**Affiliations:** 1 Universitat Rovira i Virgili; Department of Psychology Centre de Recerca en Avaluació i Mesura de la Conducta Institut d'Investigació Sanitària Pere Virgili Tarragona Spain

**Keywords:** mHealth, mobile health, mobile apps, evaluation studies, rating, interrater reliability, MARS, MAG

## Abstract

**Background:**

There is a huge number of health-related apps available, and the numbers are growing fast. However, many of them have been developed without any kind of quality control. In an attempt to contribute to the development of high-quality apps and enable existing apps to be assessed, several guides have been developed.

**Objective:**

The main aim of this study was to study the interrater reliability of a new guide — the Mobile App Development and Assessment Guide (MAG) — and compare it with one of the most used guides in the field, the Mobile App Rating Scale (MARS). Moreover, we also focused on whether the interrater reliability of the measures is consistent across multiple types of apps and stakeholders.

**Methods:**

In order to study the interrater reliability of the MAG and MARS, we evaluated the 4 most downloaded health apps for chronic health conditions in the medical category of IOS and Android devices (ie, App Store and Google Play). A group of 8 reviewers, representative of individuals that would be most knowledgeable and interested in the use and development of health-related apps and including different types of stakeholders such as clinical researchers, engineers, health care professionals, and end users as potential patients, independently evaluated the quality of the apps using the MAG and MARS. We calculated the Krippendorff alpha for every category in the 2 guides, for each type of reviewer and every app, separately and combined, to study the interrater reliability.

**Results:**

Only a few categories of the MAG and MARS demonstrated a high interrater reliability. Although the MAG was found to be superior, there was considerable variation in the scores between the different types of reviewers. The categories with the highest interrater reliability in MAG were “Security” (*α*=0.78) and “Privacy” (*α*=0.73). In addition, 2 other categories, “Usability” and “Safety,” were very close to compliance (health care professionals: *α*=0.62 and 0.61, respectively). The total interrater reliability of the MAG (ie, for all categories) was 0.45, whereas the total interrater reliability of the MARS was 0.29.

**Conclusions:**

This study shows that some categories of MAG have significant interrater reliability. Importantly, the data show that the MAG scores are better than the ones provided by the MARS, which is the most commonly used guide in the area. However, there is great variability in the responses, which seems to be associated with subjective interpretation by the reviewers.

## Introduction

In recent years, there has been an explosion of interest in the use of mobile devices (eg, smartphones, tablets) [1], alongside huge advances in the development of health-related mobile apps [2]. For example, a total of 325,000 different health-related apps has recently been reported to be available [3]. There are mobile apps for virtually all kinds of health conditions: for example, chronic pain [4,5], cancer [6], diabetes [7], and cardiovascular diseases [8]. This growth has brought considerable benefits not only to patients but also to society at large and at multiple levels. For example, health-related apps help to (1) improve treatment management, (2) facilitate patient-doctor communication, (3) monitor the patient's condition in real time, and (4) improve accessibility to treatment [9-12]. But there is also a number of caveats, mostly related to the somewhat unsupervised and unregulated nature of the process. And it has been suggested that the fact that the field is evolving without much scientific support or guidance [13] not only acts as a barrier to improvement [14] but also, and more importantly, can potentially put an individual’s health at risk [15]. Some of the main problems related to health apps are (1) faulty reminders that make proper treatment follow-up difficult (eg, the instructions on when to do an activity or take medication are not correct [16]); (2) lack of health expert involvement [17]; (3) inappropriate response to consumer needs (eg, bipolar disorder apps failing to provide any response when asked about extreme mood swings or suicidal ideation [18]); and (4) incorrect medication doses (eg, incorrect calculation of insulin dose from blood glucose values [19]).

In order to overcome the issues health-related apps are facing, some rating scales and guides have been developed (eg, [20,21]). One of the first was the Mobile App Rating Scale (MARS) [22]. It is one of the most used rating scales to measure the quality of health-related apps [23-27]. However, the MARS was created from a narrow perspective [28-30] on the basis of analyzing studies on existing mobile apps and leaving out information from other relevant sources (eg, standards governing the design of software for medical devices).

Recently, the Mobile App Development and Assessment Guide (MAG) [13] was created to address the problems observed in the guides available (but not current key concerns such as privacy and security) and to help assess health-related apps and guide stakeholders in the development of new quality apps. The MAG was developed using data from all potential relevant sources and a representative sample of the guidelines, frameworks, and standards in the field of health app development. The MAG has been acknowledged as a good quality guide by an international and interdisciplinary group of stakeholders [31].

These guides are important in the field as they provide quality scores that are key to identifying the best apps available and distinguishing them from the poorly designed ones. However, there are little data on the comparative value and consistency of the very few guides there are. The field would benefit considerably from studies that guide the development of new apps and comparatively assess the quality of existing ones.

The main objective of this research was to study and compare the MAG and MARS. More specifically, we aimed to compare the interrater reliability of the 2 measures. We also focused on whether the interrater reliability of the measures is consistent across multiple types of apps and stakeholders.

## Methods

### App Selection Process

In order to evaluate the interrater reliability of the MAG and MARS across different types of apps, we evaluated the top 4 search results for chronic health conditions in the medical category of the Apple and Android stores (ie, App Store and Google Play, respectively). The search and selection of the apps were conducted in October 2020.

The inclusion criteria were as follows: The app had to be focused on a chronic health condition, in English or Spanish, and free to download. We selected chronic health conditions because it is one of the domains in which health apps are becoming more relevant (56% of health apps are intended for this kind of patient [32]). Reports by governmental agencies indicate that chronic health conditions are a major health problem that affects 31% of the population [33-36]. In addition, chronic health conditions are the leading cause of death and disability in both the developed and developing world in the global burden of disease equation. The most important chronic health conditions are low back pain and headache, neoplasms, diabetes and kidney diseases, and cardiovascular diseases [37-40]. We used the following search terms, which are related to the top 4 chronic health conditions in the Global Burden of Disease study [41]: “pain,“ “cancer,” “diabetes,” and “cardiovascular.” In this search, we identified 886 apps and excluded 265 as they were not related to any of the 4 health conditions of interest. Finally, we selected the top 4 most downloaded apps (1 for each chronic health condition), which we then used in this study.

### App Evaluation Process

The apps were rated by 8 reviewers during the months of October and November 2020. The reviewers were a group of stakeholders that included clinical researchers, engineers, health care professionals, and end users as potential patients. These groups of stakeholders were identified as representative of individuals that would be most knowledgeable and interested in the use and development of health-related apps. The individuals in the “end users/potential patients” and “health care professionals” groups were identified and approached by the authors while at the university hospital (for a health checkup or while at work, respectively). The individuals in the “clinical researchers” and “engineers” groups were professors or technicians working at the university. Only individuals that agreed to participate and reported having experience in the use of smartphones and health apps were selected. All individuals approached were included. Reviewers received (1) the list of apps, (2) a survey including the items of the MAG and MARS to be evaluated, and (3) specific instructions as to how to proceed with the review and evaluation of the apps. In order to avoid potential interferences and help reviewers to work independently, and in line with similar studies (eg, [42]), they were not given any other suggestions, indications, or training about the procedure.

For the evaluation, all reviewers downloaded and installed the apps on their personal mobile device. Then, they reviewed each of the apps using the specific criteria in the MAG and MARS. In their assessment, the reviewers were instructed to only take into account the content and information provided within the app itself and the stores (ie, App Store and Google Play). This included websites, scientific studies, and other external references as long as they were suggested or mentioned explicitly within the app or the stores. Like similar successful procedures, the reviewers did not receive any specific training, and although they spent several minutes examining the apps, they were not instructed to use them realistically [42]. The objective of this activity and procedure was that they would evaluate the apps in the same way as experts who do not need them would.

The MAG [31] has 48 items grouped into 8 categories or domains: usability, privacy, security, appropriateness and suitability, transparency and content, safety, technical support and updates, and technology. The reviewers used each of the items in the categories to assess the quality of the apps and checked if the apps met those characteristics and functions (1=yes; 0=no).

The MARS [22] has 23 items that are grouped into 5 categories: engagement, functionality, aesthetics, information quality, and subjective quality. It also has 6 items that are app-specific and can be adapted to include or exclude specific information on the topic of interest. For example, these items have been used to assess the perceived effects on the user’s knowledge, attitudes, and intentions to change as well as the likelihood of changing the identified targeted behaviors in a study of mobile apps supporting heart failure symptom monitoring and self-care management [23]. In this study, we discarded these app-specific items. When using the MARS, the reviewers used each of the items to assess the quality of the apps and scored them using a 5-point rating scale (1=inadequate, 2=poor, 3=acceptable, 4=good, 5=excellent).

### Data Analysis

In order to study and compare the interrater reliability of the MAG and MARS, we calculated the Krippendorff alpha [43,44] for every category in the 2 guides, for each kind of reviewer and every app, separately and combined. The Krippendorff coefficient has been found to be superior to the Cohen coefficient and can be used with an unlimited number of reviewers [45-47]. An alpha ˃0.667 has been identified as showing acceptable agreement [44]. Therefore, in this study, we used this figure as the minimum level showing agreement [44]. A negative alpha indicated that agreement was less than could be expected by chance. All data analyses were performed using SPSS v.26 for Windows using the Kalpha macro [48].

## Results

A total of 8 reviewers rated the 4 apps using the MAG and MARS guides. The mobile apps included in the analysis were “Manage My Pain” (ie, pain), “BELONG Beating Cancer Together” (ie, cancer), “mySugr - Diabetes App & Blood Sugar Tracker” (ie, diabetes), and “ASCVD Risk Estimator Plus” (ie, cardiovascular diseases).

The group of reviewers included 2 clinical researchers, 2 engineers, 2 health care professionals, and 2 end users as potential patients. Reviewers’ ages ranged from 24 to 40 years old, with an equal distribution of women and men. Clinical researchers, engineers, and health care professionals had been involved in the development of health-related apps, but not in any of the apps and guides used in this study (they did not have any conflicts of interest). All reviewers were highly educated individuals (all had completed university studies) and were experienced smartphone and mobile app users.

Complete responses were provided for almost all criteria and apps, although a small number of criteria showed a percentage of data completeness that ranged from 78% to 97% (eg, “It has password management mechanisms”; see Multimedia Appendix 1). Tables 1 and 2 show the interrater reliability coefficients by categories and overall for both guides.

**Table 1:** Interrater reliability scores when reviewers used the Mobile App Development and Assessment Guide (MAG).

Category	Reviewers				
	Clinical researchers	Engineers	Health care professionals	End users	Aggregate
Usability	0.28	0.28	0.62	0.45	0.38
Privacy	0.36	0.73	0.42	0.43	0.45
Security	0.18	0.78	0.76	0.26	0.47
Appropriateness and suitability	0.38	0	–0.15	0	0.25
Transparency and content	0	1	–0.40	–0.36	0.15
Safety	0.59	0.51	0.61	–0.23	0.33
Technical support and updates	0.38	1	1	0.76	0.30
Technology	0.44	0.45	–0.05	0.45	0.39
Total	0.40	0.66	0.55	0.29	0.45

**Table 2:** Interrater reliability scores when reviewers used the Mobile App Rating Scale (MARS).

Category	Reviewers				
	Clinical researchers	Engineers	Health care professionals	End users	Aggregate
Engagement	0.18	0.50	0.53	0.41	0.43
Functionality	0.24	0.52	0.40	–0.38	0.19
Aesthetics	0.42	0.26	0.23	–0.14	0.17
Information	0.03	0.08	0.05	–0.09	0.06
Subjective	0.57	0.41	–0.08	0.54	0.43
Total	0.27	0.41	0.25	0.19	0.29

For the MAG, the reviewers’ scores for several categories complied with the criteria. The highest interrater reliability scores were for the categories “Privacy” (engineers: *P*=.73) and “Security” (engineers: *P*=.78; health care professionals: *P*=.76). In addition, 2 other categories, “Usability” and “Safety,” were very close to compliance (health care professionals: *P*=.62 and *P*=.61, respectively). The total interrater reliability of MAG (ie, for all categories) was 0.45 (see Table 1).

For the MARS, none of the reviewers’ scores or the aggregate scores complied with the criteria. The categories with the highest interrater index were “Engagement” and “Subjective” with an overall alpha coefficient of 0.43 in both cases. The total interrater reliability of the MARS (ie, for all categories) was 0.29 (see Table 2).

Tables 3 and 4 show the interrater reliability scores for each mobile app assessed using the MAG and MARS guides. As can be seen, none of the scores complied with the criteria overall or in any category. Nevertheless, the highest interrater reliability scores were for the MAG guide.

A comparison of the interrater reliability between MAG and MARS is shown in Table 5. Additional supplementary information is also provided on the interrater reliability scores for each item (see Multimedia Appendix 1).

**Table 3:** Interrater reliability scores for apps when reviewers used the Mobile App Development and Assessment Guide (MAG).

Category	Mobile apps			
	Manage My Pain	BELONG Beating Cancer Together	mySugr - Diabetes App & Blood Sugar Tracker	ASCVD Risk Estimator Plus
Usability	0.58	0.49	0.27	0.15
Privacy	0.47	0.38	0.28	0.20
Security	0.44	0.18	0.42	0.32
Appropriateness and suitability	1	0.42	0	–0.04
Transparency and content	0.08	–0.08	–0.06	0.00
Safety	0	0.47	0.33	0.21
Technical support and updates	0.10	0.57	0.16	0.10
Technology	0.17	0.36	0.12	0.45
Total	0.53	0.42	0.32	0.35

**Table 4:** Interrater reliability scores for apps when reviewers used the Mobile App Rating Scale (MARS).

Category	Mobile apps			
	Manage My Pain	BELONG Beating Cancer Together	mySugr - Diabetes App & Blood Sugar Tracker	ASCVD Risk Estimator Plus
Engagement	0.31	0.24	–0.10	0.18
Functionality	0.27	0.05	–0.02	0.16
Aesthetics	–0.05	–0.03	–0.07	0.12
Information	–0.08	0.08	–0.03	0.09
Subjective	0.55	0.44	0.16	0.14
Total	0.20	0.18	0.01	0.42

**Table 5:** Interrater reliability scores of the Mobile App Development and Assessment Guide (MAG) and the Mobile App Rating Scale (MARS).

Guide and category		Reliability
**MAG**		
	Usability	0.38
	Privacy	0.45
	Security	0.47
	Appropriateness and suitability	0.25
	Transparency and content	0.15
	Safety	0.33
	Technical support and updates	0.30
	Technology	0.39
	Total	0.45
**MARS**		
	Engagement	0.43
	Functionality	0.19
	Aesthetics	0.17
	Information	0.06
	Subjective	0.43
	Total	0.29

## Discussion

### Principal Findings

This research is the first to measure the interrater reliability of the MAG [13,31]. We used the MAG to study 4 mobile health–related apps and compared the results with those obtained with the MARS [22], one of the most extensively used guides in the field.

In studies using the Krippendorff alpha, it is customary to require an alpha >0.800. However, an alpha ˃0.667 has been identified as indicative of acceptable agreement, and anything below that is considered as unacceptable [42,44]. The data revealed that few categories reached that score and showed high interrater reliability. This finding is similar to that of other studies (eg, [26,42,46]) that have analyzed this type of guides. Taken as a whole, the findings demonstrate that it is difficult for reviewers to rate the apps in the same or similar way. First of all, reviewers greatly differed in the amount of time spent in reviewing each app (ranging from 30 minutes to 60 minutes). Thus, it is possible that the time spent in the review process had an influence on the results of the assessment. Our data did not show differences associated to the amount of time spent in the review. However, we used a small number of reviewers (n=8). Therefore, additional research to study this issue is warranted. Another potential explanation for the findings is that reviewers do not interact with the apps in the same way, so they display different responses and functions [46]. Therefore, it is unlikely that reviewers will detect all app functions, which leads to differences in the ratings because they might not be assessing exactly the same items. Support for this explanation can be found in the fact that the most objective categories evaluated, those which require less subjective interpretation by the reviewer (eg, “Privacy,” “Security”), are the ones with the highest interrater reliabilities. This finding is similar to the one reported by Powell and colleagues [42], who detected that the less judgment required by reviewers, the higher the reliability.

Another important finding of this study was that interrater reliability scores for the MAG were better than for the MARS. Importantly, some of the MAG categories with the highest interrater reliability are not included in the MARS (eg, “Privacy,” “Security,” “Technology”). These are issues that have grown in importance in the field in recent years.

It should also be noted that some MAG categories showed a higher interrater reliability than others, but there was considerable variation in the scores between the types of reviewer. This finding suggests that the differences in the interrater reliability scores are related to such individual characteristics of the reviewers as background or training. This could help explain, in part at least, why engineers showed the highest reliability scores in the category of “Security,” as this is an important issue that is currently a matter of key interest in the training of engineers but not in the case of clinical researchers. And it implies that reviewers from different backgrounds are required to assess apps and that reviewers need to be trained. However, it is also possible that the low interrater reliability scores were not only reviewer-related, but also app-related. That is, although we selected the 4 most downloaded apps, they may not have been quality apps or easy to assess (eg, the functions or properties of the apps were not easy to find or identify). In support of this explanation, some items were not answered by any reviewers in either of the guides (eg, “It has a data recovery system in case of loss”; “It is based on ethical principles and values”). Finally, another nonexclusive explanation for these results could be related to the guides (ie, the MAG and MARS). The fact that the categories that required less interpretation (eg, “Security”) were the ones with the highest interrater reliability would support this explanation. This suggests that the guides must be improved.

The differences in interrater reliability and more importantly, the lower scores found suggest that there is a very important underlying problem that is indicative of the difficulty of creating a good guide to help in the development and assessment of health-related apps. On the basis of the results of this study and others (eg, [42,46]), users of health-related apps should use and interpret the results of quality assessments with caution. The guides, as they are, have not been demonstrated to provide a secure reliable measure of the overall quality of the apps.

The assessment of the quality of health-related apps is very important. Therefore, we must continue working on improving the way assessments are conducted. This may not only require improving the available guides but also working with specialized centers and trained reviewers.

### Future Research

Studies are needed to help improve available guides that are psychometrically sound so future research should focus on how to improve and empirically test interrater reliability. For example, studies should examine whether giving reviewers additional training is enough or how reviewers’ knowledge and assessment skills can best be improved. They should also establish whether the quality of health-related apps should be assessed by reviewers with different qualifications, training, and background. Moreover, since subjectivity might be an issue in the guides, an area for improvement is that guides include clearly defined criteria. Therefore, research to determine whether understandable and well-defined criteria can improve interrater reliability above and beyond the improvement in reviewer training is warranted. Moreover, and specifically in relation with the MAG, additional research with more apps of different types is also warranted. This would help ascertain whether and how different types of app influence the reviewers' evaluations. In addition, the criteria and the categories included in the guide deserve specific attention. Studies with additional samples of reviewers, including individuals with chronic health conditions, to evaluate their comprehensibility and appropriateness are needed.

### Limitations

This study has a number of limitations that should be taken into account when interpreting the results. First, we studied the interrater reliability of the MAG when it was used to evaluate apps that were available for both Android and IOS. Although the apps are generally the same on both platforms, there may be small differences that influence the user’s experience or performance when using different platforms and devices. For example, the amount of information displayed or the position and size of some elements (eg, buttons, menu) may differ due to the size of the screen. Second, we used a very limited number of apps. We selected the most downloaded ones, as we thought they would be of better quality and therefore easier for reviewers to assess. However, they may not be of quality or representative of health-related apps and so may not be suitable for an accurate study of the interrater reliability of the guides. Third, during the period of time that the apps were being assessed, they may have been updated or modified, which would have had an unknown impact on the results of the assessments. Fourth, although individuals from different groups participated, they may be not representative. Even though they were extremely knowledgeable in their respective areas, they may or may not be the best individuals to assess the quality of the apps, as none of them had received any training. Moreover, they did not receive any substantial training in using the MAG or MARS. Thus, it is unclear whether the low interrater reliability is related to the instrument that is being used, to the lack of training provided to the raters, or both. We decided not to give specific training as we wanted to study whether the MAG and MARS can be reliably used as they are. Previous studies have also used this strategy (eg, [42]). However, future studies should examine whether training can help improve the reviewers’ assessment and the interrater reliability.

### Conclusions

Despite the limitations of the study, our findings provide new and important information about the MAG. Of particular consequence is that several categories in the MAG have significant interrater reliability. In addition, the data show that the scores are better than the ones provided by the MARS, the most commonly used guide in the area.

## Appendix

Multimedia Appendix 1 Interrater reliability scores and data completeness for each item.

## Abbreviations

MAG: Mobile App Development and Assessment Guide
MARS: Mobile App Rating Scale
