# Supplementary material for: Assessing the Quality of Mobile Health-Related Apps: Interrater Reliability Study of Two Guides
Source: JMIR Mhealth Uhealth. 2021 Apr 19;9(4):e26471. doi: 10.2196/26471 (PMC8094021; doi:10.2196/26471)
Supplement: Multimedia Appendix 1 [file mhealth_v9i4e26471_app1.pdf]

Interrater reliability scores and data completeness for each item

The Mobile App Development and Assessment Guide (MAG)

Category	Item	Reliability	Completeness; %
**Usability**			
	The app has been tested by potential users before being made available to the public.	0.05	100
	It is easy to use (that is, navigation is intuitive).	0.66	100
	Functionality is adapted to the purpose of the application.	0.11	100
	Functionality is adjusted according to the profile and needs of the targeted user.	0.11	100
	Access is adapted for people with disabilities.	-0.02	100
	It complies with regulatory accessibility standards.	0.13	100
	The language used makes it accessible to any user.	0.26	100
	All users have access to all resources regardless of their capabilities.	-0.09	100
**Privacy**			
	The app gives information about the terms and conditions of purchases in the application.	0.76	100
	It must only ask for user data that is essential for the application to operate.	0.03	100
	It gives information about access policies and data processing, and ensures the right of access to recorded information.	0.52	100
	It gives information about possible commercial agreements with third parties.	0.48	100
	It clearly allows the user the option of non-transfer of data to third parties or for commercial purposes.	0.80	100
	It guarantees the privacy of the information recorded.	0.45	100
	It requires users to give their express consent.	0.68	100
	It warns of the risks of using the application.	0.08	100
	It tells users when it accesses other resources on the mobile device such as their accounts or their social network profiles.	0.09	93.75
	It takes measures to protect minors in accordance with current legislation.	0.25	100
	Confidential user data is protected and anonymized, and there is a privacy mechanism so that users can control their data.	0.52	93.75
	It offers to erase the data when the service is finished.	0.12	100
	It gives information about privacy policies in a simple and understandable way.	0.34	100
	It complies with all current privacy laws.	0.11	100
**Security**			

	The app has encryption mechanisms for storing, collecting and exchanging information.	0.47	87.5
	It has password management mechanisms.	0.44	93.75
	It states the terms and conditions of cloud services.	0.26	100
	The cloud services used have the relevant security measures.	0.66	87.5
	The authorization and authentication mechanisms protect users' credentials and allow access to their data.	0.81	81.25
	It limits access to data that is only necessary for the user.	-0.02	100
	It detects and identifies cybersecurity vulnerabilities, possible threats and the risk of being exploited.	0.25	100
	It applies the appropriate security measures to cybersecurity vulnerabilities in the face of possible threats, in order to reduce the risk of being exploited.	0.12	96.88
	It informs users of the possible risks associated with the application's use of personal data.	0.20	100
**Appropriateness and suitability**			
	The benefits and advantages of using the app are explained.	0.11	100
	Experts have participated in the development of the app (for example, specialized professionals, health organizations, scientific societies or specialized external organizations).	0.27	100
**Transparency and content**			
	It uses scientific evidence to guarantee the quality of the content.	0.03	100
	It is based on ethical principles and values.	-0.08	87.5
**Safety**			
	The possible risks to users are identified.	0.05	100
	It ensures that there are no adverse effects.	-0.08	100
	It complies with regulatory standards as a medical device.	-0.01	100
	Users are warned when adverse events are identified so they can delete the application and avoid potential risks.	0.05	100
	Users are warned that the app is not meant to replace the services provided by a professional.	0.27	100
	It recommends always consulting a specialist in case of doubt.	0.38	100
	Potential risks for users caused by incorrect usage and/or possible adverse effects are explained.	0.23	100
**Technical support and updates**			
	It gives a warning if updates can influence insensitive data (changes the use of the data or different data is collected).	0.06	90.63

	Every time an update of a third-party component is published, the change is inspected and the risk evaluated.	0	100
**Technology**			
	It works correctly. It does not fail during use (blocks, etc.).	0.13	100
	Functions are correctly retrieved after context changes (switch to another app and return, etc.), external interruptions (incoming calls or messages, etc.) and switching off the terminal.	0.24	100
	It does not waste resources excessively: battery, CPU, memory, data, network, etc.	0	100
	It has a data recovery system in case of loss.	0.32	78.13

The Mobile App Rating Scale (MARS)

Category	Item	Reliability	Completeness; %
**Engagement**			
	Entertainment: Is the app fun/entertaining to use? Does it use any strategies to increase engagement through entertainment (e.g. through gamification)?	0.54	100
	Interest: Is the app interesting to use? Does it use any strategies to increase engagement by presenting its content in an interesting way?	0.43	100
	Customisation: Does it provide/retain all necessary settings/preferences for apps features (e.g. sound, content, notifications, etc.)?	0.25	100
	Interactivity: Does it allow user input, provide feedback, contain prompts (reminders, sharing options, notifications, etc.)? Note: these functions need to be customisable and not overwhelming in order to be perfect.	0.37	100
	Target group: Is the app content (visual information, language, design) appropriate for your target audience?	0.22	100
**Functionality**			
	Performance: How accurately/fast do the app features (functions) and components (buttons/menus) work?	0.26	100
	Ease of use: How easy is it to learn how to use the app; how clear are the menu labels/icons and instructions?	0.18	100
	Navigation: Is moving between screens logical/accurate/appropriate/ uninterrupted; are all necessary screen links present?	-0.06	100
	Gestural design: Are interactions (taps/swipes/pinches/scrolls) consistent and intuitive across all components/screens?	0.08	100
**Aesthetics**			
	Layout: Is arrangement and size of buttons/icons/menus/content on the screen appropriate or zoomable if needed?	-0.03	100
	Graphics: How high is the quality/resolution of graphics used for buttons/icons/menus/content?	0.03	100
	Visual appeal: How good does the app look?	0.49	100

**Information**			
	Accuracy of app description (in app store): Does app contain what is described?	-0.07	100
	Goals: Does app have specific, measurable and achievable goals (specified in app store description or within the app itself)?	0.16	100
	Quality of information: Is app content correct, well written, and relevant to the goal/topic of the app?	0.05	100
	Quantity of information: Is the extent coverage within the scope of the app; and comprehensive but concise?	0.31	100
	Visual information: Is visual explanation of concepts – through charts/graphs/images/videos, etc. – clear, logical, correct?	0.25	96.88
	Credibility: Does the app come from a legitimate source (specified in app store description or within the app itself)?	0.05	100
	Evidence base: Has the app been trialled/tested; must be verified by evidence (in published scientific literature)?	-0.11	93.75
**Subjective**			
	Would you recommend this app to people who might benefit from it?	0.06	93.75
	How many times do you think you would use this app in the next 12 months if it was relevant to you?	0.20	87.5
	Would you pay for this app?	-0.08	100
	What is your overall star rating of the app?	0.50	100
